# Abdominal aortic calcification can predict all-cause mortality and CV events in dialysis patients: A systematic review and meta-analysis

**DOI:** 10.1371/journal.pone.0204526

**Published:** 2018-09-21

**Authors:** Qingyu Niu, Yang Hong, Cho-Hao Lee, Chuncui Men, Huiping Zhao, Li Zuo

**Affiliations:** 1 Department of Nephrology, Peking University People’s Hospital, Beijing, China; 2 Urology and Lithotripsy Center, Peking University People’s Hospital, Beijing, China; 3 Division of Hematology and Oncology Medicine, Department of Internal Medicine, Tri-Service General Hospital, National Defense Medical Center, Taipei, Taiwan, Republic of China; University of Mississippi Medical Center, UNITED STATES

## Abstract

**Background:**

Abdominal aortic calcification (AAC) has a pretty high incidence in dialysis patients and may be associated with their prognosis. AAC can be assessed by abdominal CT or X-ray. We determined to investigate whether the occurrence of AAC is associated with all-cause mortality and cardiovascular (CV) events in dialysis patients through this meta-analysis and systematic review.

**Methods:**

A comprehensive literature search was conducted using the PubMed, Cochrane library, Embase, Medline databases to collect cohort studies investigating whether AAC is associated with all-cause mortality and CV events of patients, and we also searched gray articles and conferences abstracts. Meta-analysis was performed by STATA software. Pooled results were expressed as hazard ratio (HR) with corresponding 95% confidence intervals (CI). Fixed-effect models were used to pool the HR of each trial.

**Results:**

10 studies (2,724 dialysis patients) were identified. The presence of AAC was associated with increased risk for all-cause mortality among dialysis patients (HR, 2.84; 95% CI, 2.03–3.98; I^2^ = 9.8%; P = 0.354). Meanwhile, there was an association between AAC and increased risk for all CV events (fatal and non-fatal) in patients (HR, 2.04; 95% CI, 1.51–2.76, I^2^ = 44.6%; P = 0.125). 3 studies presented their endpoint as CV mortality, and the pooled HR was 2.46 (95%CI 1.38–4.40; I^2^ = 0.0%; P = 0.952).

There were also 2 studies that reported their primary endpoint as all-cause mortality and CV events, and the pooled HR was 5.72 (95% CI 3.24–10.10; I^2^ = 0.0%; P = 0.453).

**Conclusions:**

Among patients treated with dialysis, AAC is associated with adverse outcomes, including all-cause mortality and CV events (fatal and non-fatal). The abdominal X-ray or CT scan can be used as a useful added method to evaluate the patient’s calcification. This may provide reasonable data for estimating the risk of adverse events in dialysis patients, which is helpful in guiding clinical treatment and improving the prognosis of dialysis patients.

## Introduction

The risk of cardiovascular (CV) disease in patients with end stage renal disease(ESRD) is far greater than that in general population patients, and CV mortality in dialysis patients is 10–20 times that of general people [1, 2]. It is increasingly believed that the presence of vascular calcification increases the risk of CV events, and it can be considered as a predictor of CV events [1]. Computer tomography (CT) scan or X-ray could be used to detect vascular calcification and estimate the extent of calcification [3, 4]. Coronary artery calcification has been considered as an important predictor of CV events and all-cause mortality in patients with ESRD [5, 6]. According to the CT-derived coronary artery calcium scores, patients with high scores have a greater risk of future CV events than those with low scores [7, 8].

In contrast, abdominal aortic calcification (AAC) has been rarely used as a marker to diagnose atherosclerosis or a tool to assessment the risk of CV events [9]. But in some studies, AAC has been shown to be an independent risk factor for all-cause mortality or CV events in the general population [4, 10, 11]. The presence of AAC was closely related to increased risk of adverse outcomes of dialysis patients [9, 12–16]. However, studies published to date have not reached consistent conclusions regarding the presence or extent of AAC and CV or all-cause mortality in maintenance dialysis patients. In addition, the range of risk estimates varied among different studies.

Therefore, we conducted a systematic review and meta-analysis, aiming to determine whether the presence of AAC can be used as an independent predictor of all-cause mortality and CV events in dialysis patients.

## Methods

### 2.1 Study design

This meta-analysis was designed and produced based on the Meta-analysis of Observational Studies in Epidemiology (MOOSE) guidelines and the PRISMA 2009 guidelines [17]. The PRISMA checklist may be found in S1 Table. This study was not registered.

### 2.2 Search strategy

A comprehensive literature search was conducted using the PubMed, Cochrane library, Embase and Medline databases from inception to 11th April 2018, without restrictions on languages. We also searched gray literature from the website of Open Gray, conference abstracts of American Society of Nephrology (ASN) (2007–2017) and European Renal Association–European Dialysis and Transplant Association (ERA-EDTA) (2007–2018). The specific searching strategy is described in S2 Table.

### 2.3 Inclusion and exclusion criteria

Trials were ensured based on the following inclusion criteria: (1) original cohort studies; (2) patients with ESRD treated with hemodialysis (HD) or peritoneal dialysis (PD); (3) reporting the relationship between the presence or extent of AAC and CV events, CV mortality or all-cause mortality risk; (4) providing risk estimate [(hazard ratios (HR) with corresponding 95% confidence intervals (CI)) of CV events, CV mortality or all-cause mortality. For the multiple articles from the same study, we only included the most recent eligible one. We excluded animal studies, conference abstracts, cross-sectional studies, case reports, reviews, and editorials.

### 2.4 Data extraction and quality assessment

The data extraction process was applied blindly. Two researchers (Qingyu NIU and Yong HONG) independently extracted the following information from each study: first author’s name, publication year, publish journals, region, study design, baseline characteristics of patients, methods used to detect calcification, scoring methods, event numbers, fully adjusted HR and 95%CI, covariates adjusted and follow-up duration. Any existing disagreement was resolved by discussion with a third reviewer (Huiping ZHAO). A kappa statistic calculated for measuring agreement between two authors making simple inclusion/exclusion decisions. The kappa value was 0.83, which between 0.40 and 0.59 have been considered to reflect fair agreement, between 0.60 and 0.74 to reflect good agreement and 0.75 or more to reflect excellent agreement [18] (S3 Table). The quality of selected studies was estimated with the Risk of Bias in Nonrandomized studies of Interventions (ROBINS-I) tool, which is a tool for assessing risk of bias in non-randomised studies of interventions [19].

### 2.5 Calcification scores

Most of the included trials were using AAC score as scoring method [9, 12, 14–16, 20–23]. AAC score was first described by Kauppila et al [4, 15]. Lateral lumbar spine radiographs were obtained to measure the severity of calcification in the aorta at the level of the first to fourth lumbar vertebrae. According to the calcification of the anterior and posterior aortic walls, 0 to 24 points was given. 1 study were using aortic calcification index (ACI) to assess the extent of AAC [24]. In this method, the abdominal aorta was studied by CT scan in consecutive sequential 8-mm slices, and the ACI was estimated as the proportion of aortic circumference covered by calcification [25]. Three studies described results as the presence or absence of AAC [9, 16, 21], four studies divided patients into two groups (high vs. low) according to their mean value of AAC scores [14, 22–24], and three presented results in tertiles according to AAC scores [12, 15, 20] (Table 1).

**Table 1:** Characteristics of the included trials and participants.

Study/year	Region	Design	Patients	Sample size (% men)	Age (Mean)	Detection Methods	Scoring Methods	Comparison of AAC	Events and HR (95% CI)				Follow-up (months)	Adjustment for Covariates	Risk of bias according to ROBINS-I
									All death	CV events (fatal and non-fatal)	All death and CV events	CV death			
**Makela2018** [12]	Sweden, Finland, Denmark, Estonia	Prospective	PD	249 (66.7)	61	Plain lateral lumbar X-ray	AAC-24	Grade3(7–24) VS. Grade2(1–6) VS. Grade1(Absence)	4.85 (1.09–21.63); 2.22 (0.44–11.18)	2.59 (1.00–6.72); 2.30 (0.84–6.34)			46	age, gender, BMI, DM, ABI, ALB	Moderate
**Wang2017** [23]	China	Prospective	HD	170	NS	lateral abdominal radiograph	AAC-24	High VS. low (AAC≥5 VS. AAC<5)	4.373 (1.562–7.246)						Moderate
**Rroji2017** [22]	Albania	NS	PD, HD	126	62.6	Lateral lumbar spine radiograph	AAC-24	High VS. low (AAC≥7 VS. AAC<7)				2.25 (1.77–5.58)			Moderate
**NasrAllah2016** [9]	Egypt	Prospective	HD	93 (48.3)	42.7	Lateral lumbar spine radiographs	AAC-24	Presence VS. absence	1.2 (0.4–4)				46.8	NS	Moderate
**Kwon2014** [14]	Korea	Retrospective	HD	112 (43.5)	59	left lumbar spine radiograph	AAC-24	High VS. low (mean AAC 8)	4.205 (1.658–10.669)	1.801 (1.281–2.531)			32.8	CCI score, ESRD&Hurations, Coronary score, CRP, Ca, LDL, iPTH	Low
**Yoon2013** [24]	Korea	Retrospective	PD	92 (52.2)	55	abdominal CT scan	ACI	High VS low (mean AAC18.9)			5.25 (1.77–15.58)		35.3	age, DM, pre-CVD, HB, ALB, CRP, LAD, ejection fraction	Moderate
**Martino2013** [20]	Italy	Prospective	PD	72 (60.8)	NS	left lateral plain radiograph	AAC-24	Grade3 (>12) VS Grade2(6–12) VS Grade1(<6)		30.7 (3.562–264.841); 3.918 (0.419–36.668)			30.5	age, urine output	Serious
**Hong2013** [21]	China	Retrospective	HD	217 (49.8)	60	lateral abdominal radiograph	AAC-24	Presence VS. absence	4.47 (1.55–12.92)			2.86 (0.93–8.81)	26	age, DM, P, ALB, HP, Kt/V, PP	Moderate
**Verbeke2011** [15]	European	Prospective	PD, HD	1076	61.9	plain lateral lumbar radiograph	AAC-24	Grade3(<5) VS Grade2(5–15) VS Grade1(<5)			8.640 (3.528–21.158); 3.682 (1.356–9.997)		24	age, DM, ALB	Moderate
**Okuno2007** [16]	Japan	cohort study	HD	515 (59.4)	60.1	left lateral abdomen radiograph	AAC-24	Presence VS. absence	2.07 (1.21–3.56)			2.39 (1.01–5.66)	51	age, HD duration, DM, BMI, ALB, CRE, P, CRP	Moderate

***Abbreviations*:** HR, hazard ratio; HD, hemodialysis; PD, peritoneal dialysis; AAC, abdominal aortic calcification; ACI, aortic calcification index; ACAI, aortic calcification area index; BMI, body mass index; DM, diabetes mellitus; ABI, Ankle-brachial index; ALB, albumin; NS, Not stated; CCI score, Charlson comorbidity index; CRP, C-reactive protein; LDL, Low Density Lipoprotein; CV events, cardiovascular events; pre-CVD previous cardiovascular disease; HB, hemoglobin; LAD, left atrial diameter; P, phosphorus; HP, hypertension; PP, pulse pressure; SP, systolic pressure; Lp (a), lipoprotein a); CRE, creatinine.

### 2.6 Outcome measures

The primary outcome was all-cause mortality. There are 3 secondary outcomes, including CV events (fatal and non-fatal), CV mortality and all-cause mortality and CV events. Several studies presented multiple end points, so we pooled them in more than one analysis to avoided the unacceptable heterogeneity resulted by combining different types of end points. The definition of non-fatal CV events not the same among different studies but generally included acute coronary syndrome, myocardial infarction, stroke and peripheral vascular disease. In each study, results that adjusted for the maximum covariates were used.

### 2.7 Statistical methods

As the homogeneity among the pooled studies was good (the I^2^ statistic <50%, Cochrane Q test P < 0.10), this meta-analysis was performed using the fixed-effect model. If the heterogeneity was obvious, The Hartung-Knapp-Sidik-Jonkman method for random effect model will be used as IntHout et al described previously [26]. The association between AAC and all-cause mortality and CV events was computed as a summary HR with 95% CI. Statistical heterogeneity among studies was assessed using the Cochrane Q test and I^2^ statistic. The significance of the statistical heterogeneity was set at the I^2^ statistic ≥50% and/or Cochrane Q test P < 0.10. Funnel plot was used to test the publication bias of pooled results. Sensitivity analysis was performed by excluding any single study to test the robustness of the pooled results. All statistical analyses were performed using STATA software (version 14.0). All tests were 2-tailed, and P <0.05 was considered statistically significant.

## Results

### 3.1 Characteristics and quality of included studies

**Fig 1:**
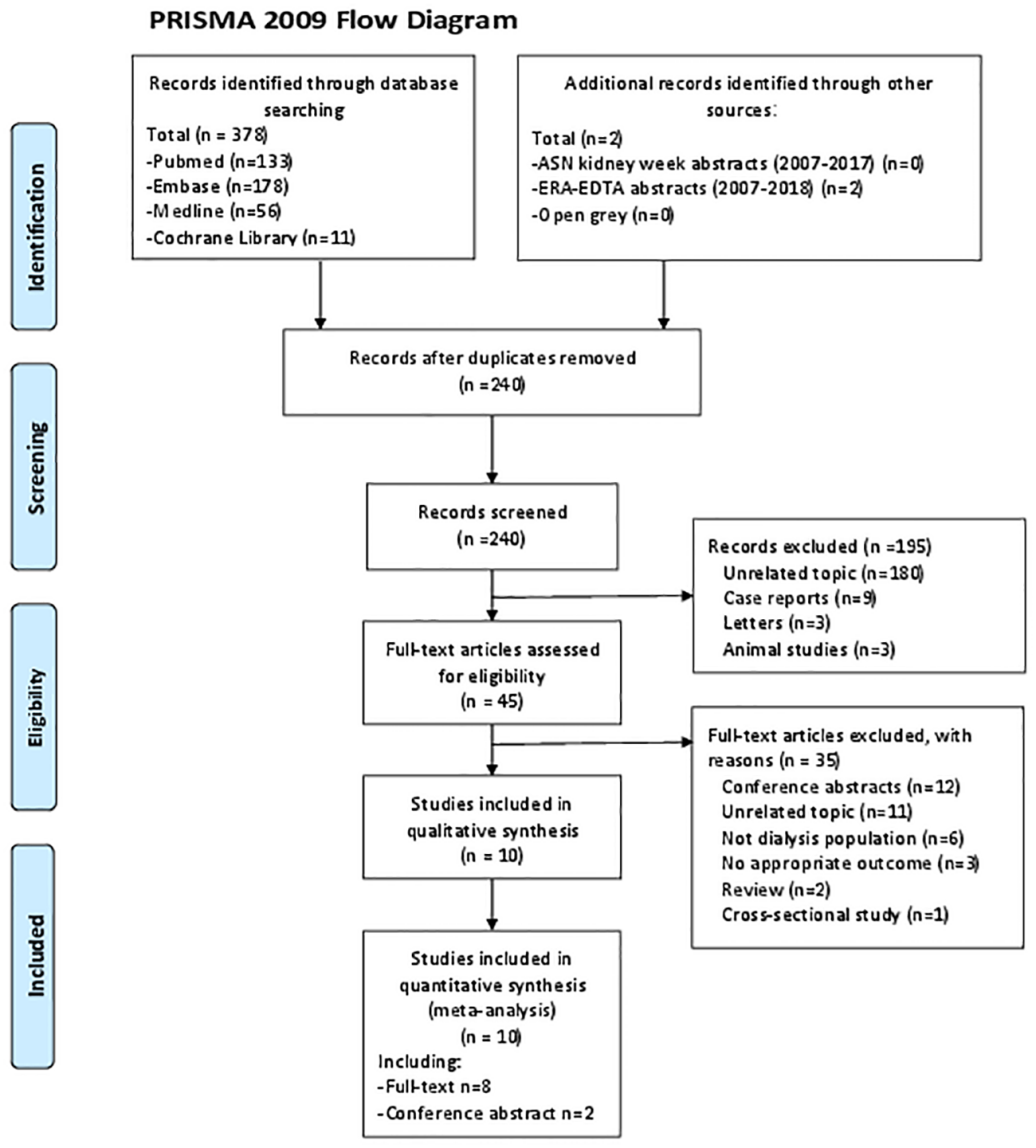
Flow chart of the identification process for eligible studies.

The search strategy identified 378 full texts in databases. We also searched gray literature from the website of Open Gray, conference abstracts of ASN and ERA-EDTA (2007–2018), then included 2 abstracts which are met our inclusion criteria. After excluding duplicates, 240 records remained. Titles and abstracts of these records were screened for inclusion. Then 195 articles were excluded due to irrelevance of topics and discrepancy in study types. Full texts of 45 records were read, then 10 cohort studies (including 2 conference abstracts) met the inclusion criteria [9, 12, 14–16, 20–24, 27]The procedure of selecting and reasons for exclusion of studies is shown in Fig 1. The study of Ohya et al [28] and Yoon et al [27] were excluded because the variables for AAC included in the COX regression analysis in their studies were continuous variables. In Ohya’s study, they presented that per 1% increase of abdominal aortic calcification index will increase risk of CV mortality by 3%. In Yoon’s study, they have shown that per 1% increase of abdominal aortic calcification index will increase risk of all-cause mortality and non-fatal CV events by 3.2%. But the variables for AAC of the studies included in the present meta-analysis were categorical variables, combining the HR and 95% CI of these studies with Ohya’s and Yoon’s studies will result in large heterogeneity.

**Fig 2:**
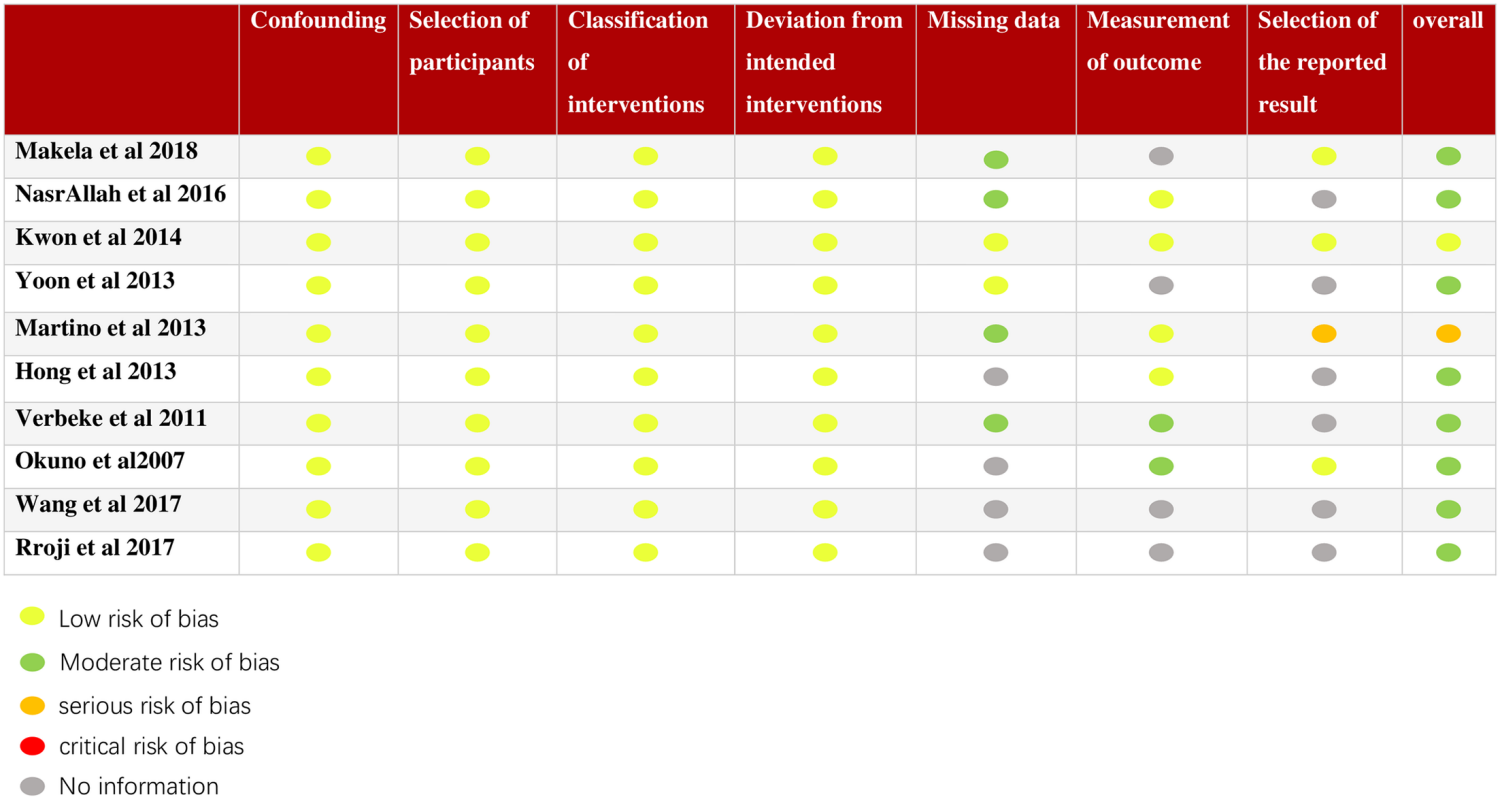
Detailed quality assessment of included studies.

Baseline characteristics and quality of selected studies assessing by ROBINS-I are listed in Table 1. 5 studies [9, 14, 16, 21, 23] enrolled patients on HD, 3 studies [12, 20, 24] enrolled patients on PD, and 2 studies [15, 22] included both. Study sample sizes ranged from 74 to 1,076, and mean follow-up duration varied from 25.2 to 95.3 months. The mean age of patients was between 42.7 and 62.6 years. Except for 1 study that was assessed as low risk of bias and 1 study that was assessed as serious risk of bias, the rest of studies were moderate. The overall assessment of the risk of bias of each study is shown in Table 1, and the detailed assessment of each study can be seen in Fig 2.

### 3.2 Association of AAC with All-cause mortality

**Fig 3:**
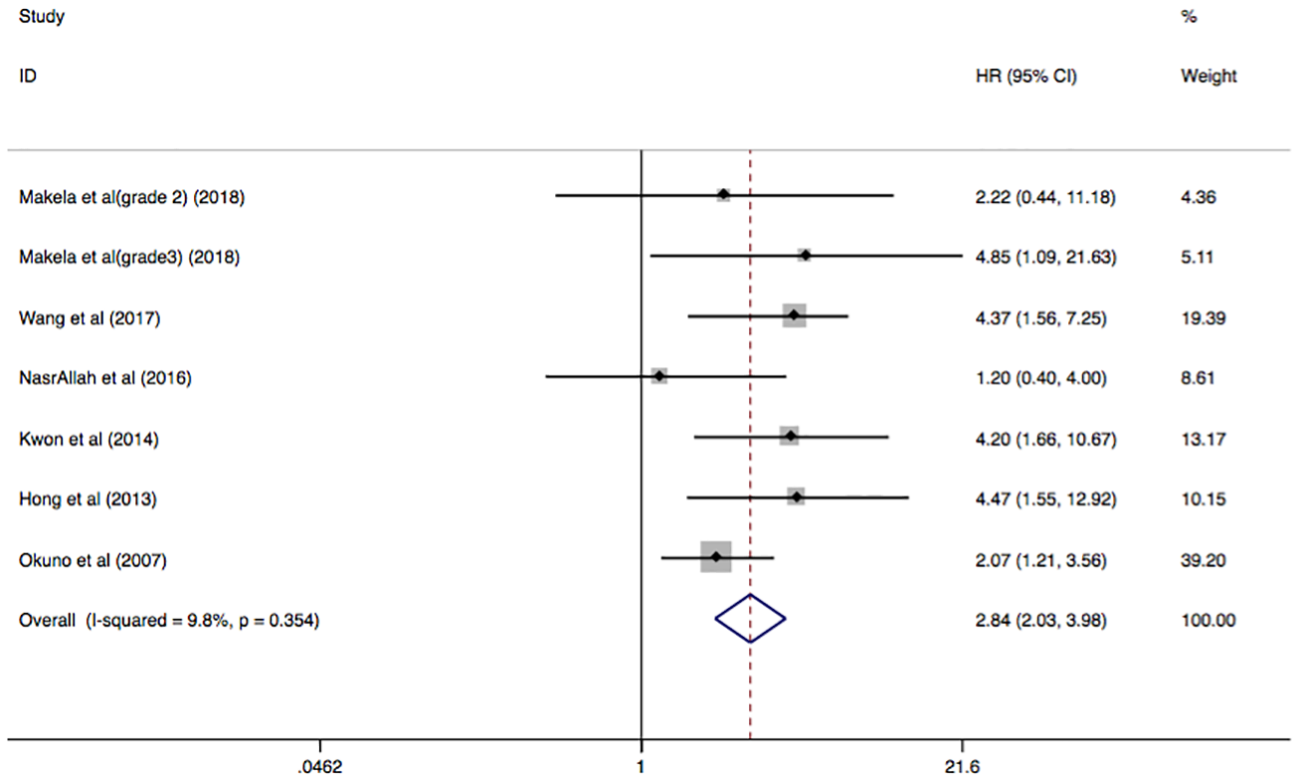
Forest plots showing HR and 95% CI of all-cause mortality compared with and without abdominal aortic calcification in a fixed effect model. HR, hazard ratio; CI, confidence interval.

All-cause mortality was reported in 6 studies, comprising analysis of 1, 356 participants. The presence of AAC was associated with increased risk of all-cause mortality among ESRD patients receiving dialysis (HR, 2.84; 95% CI, 2.03–3.98; I^2^ = 9.8%; P = 0.354) (Fig 3).

### 3.3 Association of AAC with secondary outcomes

**Fig 4:**
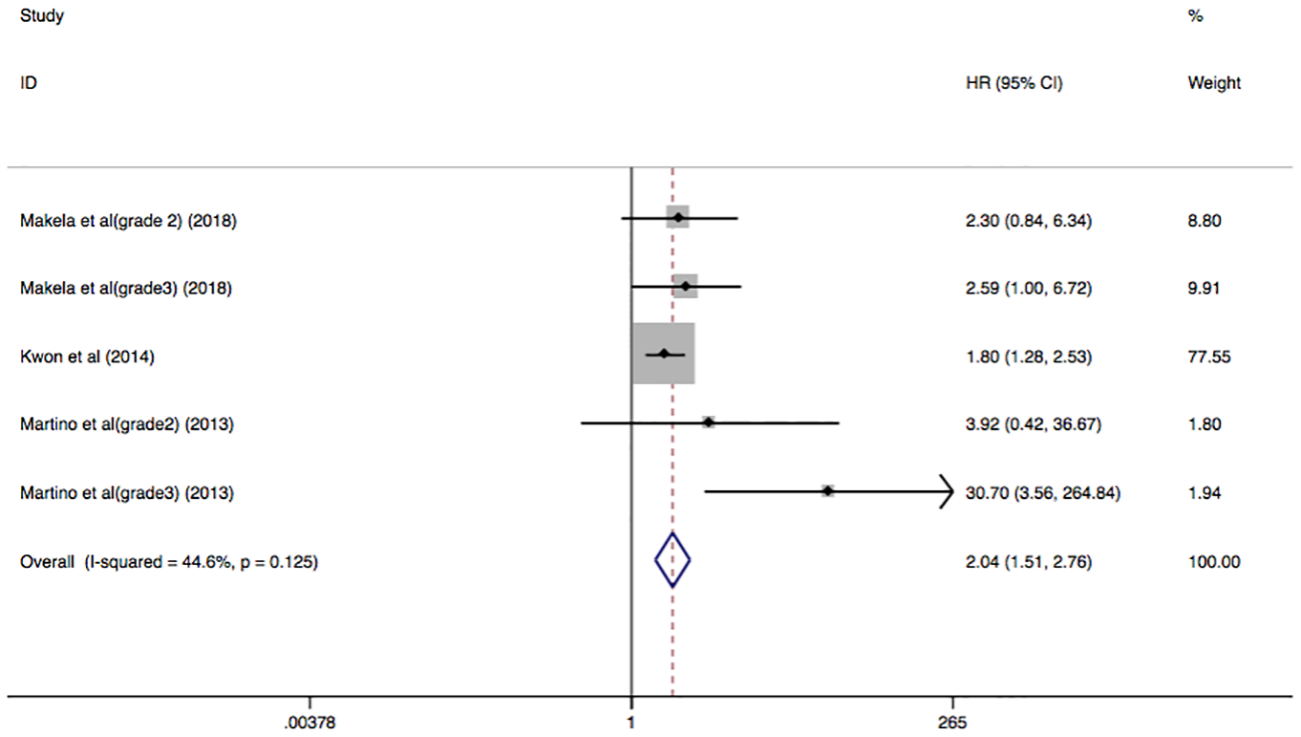
Forest plots showing HR and 95% CI of all CV events (fatal and non-fatal) compared with and without abdominal aortic calcification in a fixed effect model.

The HR of CV events (fatal and non-fatal) was reported in 3 studies with 435 participants. There was an association between the presence of AAC and increased risk for all CV events in patients with ESRD (HR, 2.04; 95% CI, 1.51–2.76) with low heterogeneity in a fixed model (I^2^ = 44.6%; P = 0.125) (Fig 4).

**Fig 5:**
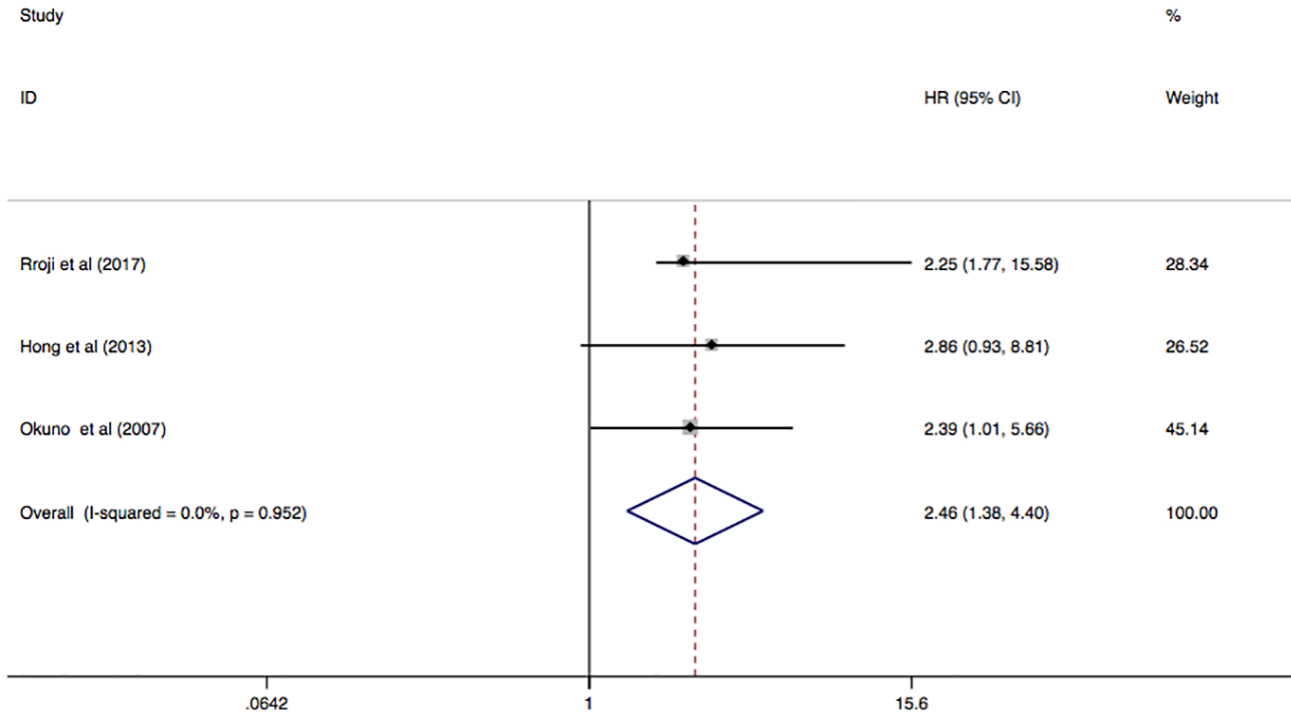
Forest plots showing HR and 95% CI of CV mortality compared with and without abdominal aortic calcification in a fixed effect model.

The pooled result of CV mortality was included 3 studies with 858 dialysis patients. This result pooled in a fixed model shown that there was a high risk of CV mortality to dialysis patients with AAC (HR 2.46; 95%CI 1.38–4.40; I^2^ = 0.0%; P = 0.952) (Fig 5).

**Fig 6:**
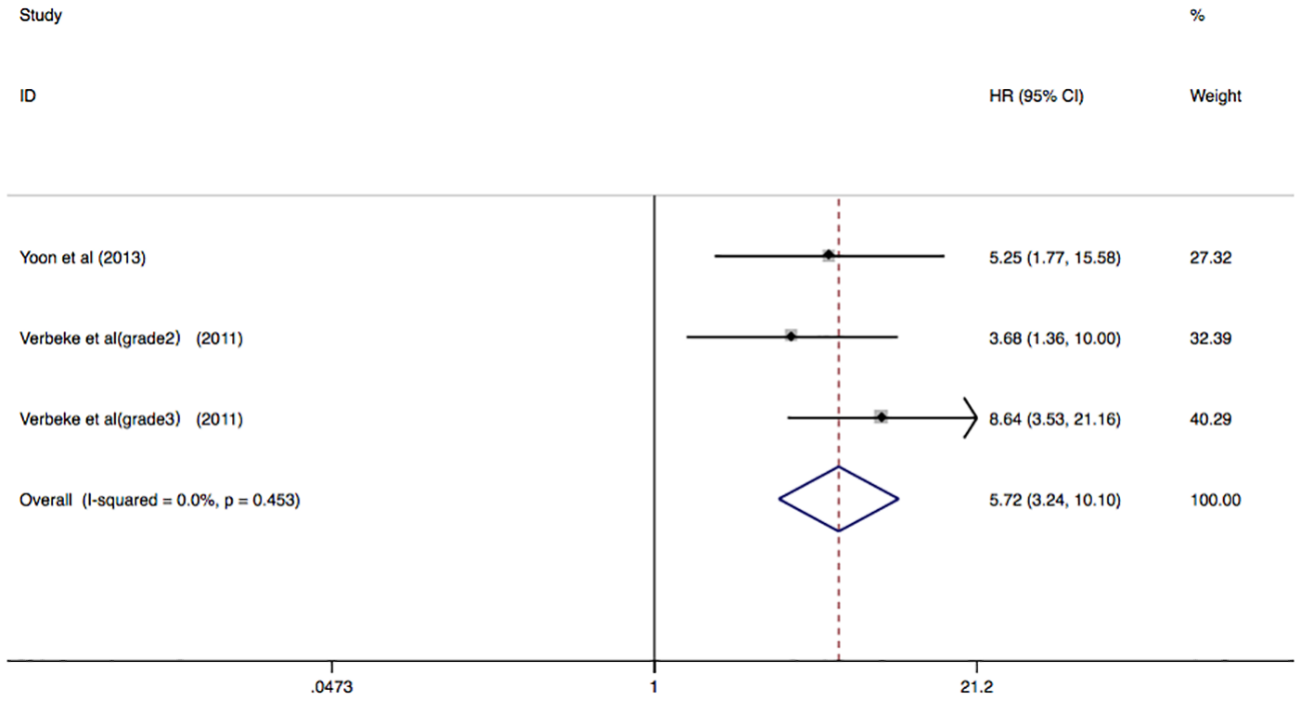
Forest plots showing HR and 95% CI of all-cause mortality and CV events compared with and without abdominal aortic calcification in a fixed effect model.

There were also 2 studies with total 1168 patients on PD or HD reported their primary endpoint as all-cause mortality and CV events. The presence of AAC was associated with greater risk of all-cause mortality and CV events (HR 5.72; 95% CI 3.24–10.10; I^2^ = 0.0%; P = 0.453) in a fixed effect model (Fig 6).

### 3.4 Publication bias and sensitivity analysis

**Fig 7:**
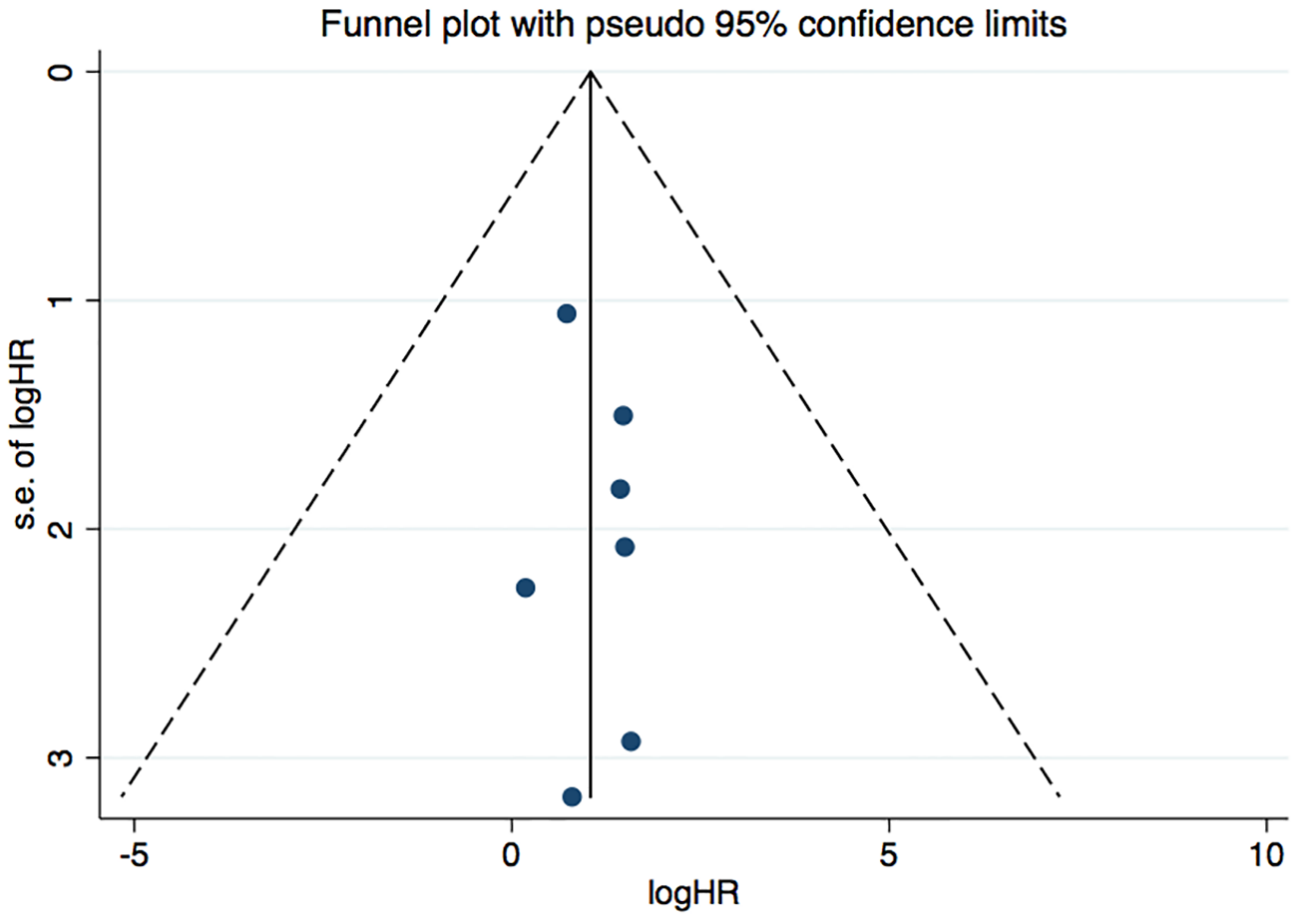
Funnel plot showing publication bias based on the all-cause mortality.

We analyzed funnel plots for every endpoint to assess the potential publication bias of the included studies. In regards to the all-cause mortality, we initially found that funnel plot yielded no statistical heterogeneity (Fig 7). Evidences of publication bias for CV events (fatal and non-fatal), CV mortality and all-cause mortality and CV events were not observed based on the funnel plot (S1, S2 and S3 Figs). We didn’t analyzed Egger’s test or Begg’s test for outcomes, since there are only 10 included studies and Egger’s test or Begg’s test may not be powered to detect publication bias.

Sensitivity analysis was performed to evaluate the stability of our results. In sensitivity analysis, excluding any single study at each turn did not lead to a change in the overall HR of all-cause mortality and other outcomes, indicating that our results were stable. (S4, S5, S6 and S7 Figs).

## Discussion

Since CV diseases are usually asymptomatic until major events occur, such as angina, heart failure and so on, the prevention of CV disease is a significant challenge to numerous patients [29]. Vascular calcification is thought to be an important cause of CV disease, and is a pretty common complication in patients with ESRD [30]. Previous studies have shown the value of evaluating vascular calcification in predicting the prognosis of dialysis patients [13, 30, 31]. Both the 2009 Kidney Disease: Improving Global Outcomes (KDIGO) guideline and the 2017 KDIGO guideline update suggested that in patients with CKD G3a–G5D, a lateral abdominal X-ray can be used to assess the presence of vascular calcification by estimating the calcification of abdominal aorta, as reasonable alternatives to CT-based imaging [32, 33]. Abdominal aorta is a susceptible site for atherosclerosis and calcification, so the presence of AAC may be related to the occurrence of CV disease and death in patients. In addition, for PD patients, assessment of AAC can provide significant information for the management of CV disease without any additional expense, because these patients have several abdominal X-ray films or CT scans to evaluate the catheter position. Therefore, it is necessary to investigate the role of abdominal aorta in predicting adverse events in patients with dialysis.

In recent years, some studies have investigated the effect of AAC on the prognosis of dialysis patients, but the results are conflicting. The study of Ohya et al [28] enrolled 137 HD patients, and they reported that AAC was not a significant prognostic factor in all-cause death (HR = 1.02, 95%CI 0.99–1.04, P value = 0.17), but AAC was useful as a prognostic indicator of CV mortality in HD patients (HR = 1.03, 95%CI 1.00–1.06, P value = 0.03). NasrAllah et al [9] found that AAC based on simple X-rays did not show a statistical association with all-cause death of patients with PD in their study. Other studies have shown that the presence of AAC is significantly associated with all-cause mortality and/or CV mortality in dialysis patients, and also recommended that careful attention should be given to the exist of AAC as a prognostic indicator [12–16, 20, 21, 24, 27]. The study of Makela et al [12] indicated that the prevalence of AAC was 81% in their prevalent PD patients, which was extremely high. Median AAC score of individual segments increased stepwise from lumbar vertebrae L1 to L4, indicating that the severity of calcification increased significantly from L1 to L4 [12]. For studies of HD patients, the prevalence of AAC was similar [15, 16].

As far as we know, the present meta-analysis is the first to determine the role of AAC in predicting all-cause mortality and CV events in dialysis patients. We included 10 studies with 2,724 participants and found that the presence of AAC was significantly associated with all-cause mortality of dialysis patients. And the patients with AAC have a higher risk of CV events (fatal and non-fatal). Based on previous studies, this study further indicated the role of evaluating abdominal aortic calcification in predicting the prognosis of dialysis patients. Hence, we should pay more attention on screening for AAC, which can be produced easily in daily clinical practice, as a prognostic indicator.

This meta-analysis provided reasonable evidence for the use of calcification of abdominal aorta as a biomarker to predict the risk of all-cause mortality and all CV events in dialysis patients. For asymptomatic patients, the classification of future risk can be based on the condition of AAC, thereby could optimize subsequent prevention strategies. In the study of Martino et al [20], they divided patients into three groups according to the AAC score: < 6 points, 6 to 12 points, and >12 points. Patients with high AAC score (>12 points) had significantly higher risk of CV events (HR 30.7, 95%CI 3.562–264.841), in comparison with those with lower AAC score (HR 3.918, 95%CI 0.419–36.668). Verbeke et al [15] investigated that AAC scores provide important predictive information for the occurrence of CV events and mortality in dialysis patients and may help to identify patients at high risk. They also divided patients into 3 groups according to the AAC score, and HR is 8.640 (95%CI 3.528–1.158) in high score group compare to 3.682 (95%CI 1.356–9.997) in moderate group. In another study, Makela et al [12] reported that severe calcification was a strong predictor of all-cause mortality (HR 4.85, 95%CI 1.09–1.63) and the occurrence of CV events (HR 2.59, 95%CI 1.00–6.72), when the extent of AAC was divided into no calcification, moderate and severe. However, the moderate calcification was not significantly related to the above adverse events (all-cause mortality HR 2.22, 95%CI 0.44–11.18; CV events HR 2.30, 95%CI 0.84–6.34). From the above discussion, it seems that as the severity of AAC increases, the mortality rate of patients may increase. It can be speculated that the classification of the severity of AAC may more appropriately predict the risk of adverse events.

There was also a meta-analysis published by Zhang et al in 2016 [34], they found that aortic arch calcification appears to be independently associated with greater risk of cardiovascular and all-cause mortality, and higher grade of AAC corresponds to a greater risk in dialysis patients. The present study looks similar to Zhang’s research, but there are differences between them. First, the calcification sites evaluated were different, they evaluated the aortic arch, and we were the abdominal aorta. Moreover, assessing the calcification of aortic arch by chest X-ray or chest CT is much difficult than that in abdominal aortic. Usually, lateral abdominal radiographs are easier to obtain than chest radiographs or chest CT scans in dialysis patients. Finally, the abdominal aorta is still a large artery, and is a site prone to atherosclerosis and calcification, which can well respond to vascular calcification in patients.

This study has several limitations. First, the protocol for our systematic review and meta-analysis was not registered publicly. For the reproducibility and validity, we provide the details of search strategy and raw data at S2 and S4 Tables. Second, the sample size of several studies was small, which may lead to limited generalizability. Third, the follow-up time of some studies was relatively short and did not exceed 3 years. Therefore, there were not enough primary end points observed, which had a certain impact on the objectivity and accuracy of the study results. Forth, different studies used different imaging methods and scoring methods to assess AAC, which made them biased in the evaluation of results. That may lead to heterogeneity when pooling risk estimates from different studies. Fifth, different covariates were adjusted in different trials, and that might have an influence on these results. Finally, some negative findings in unpublished trials that we didn’t included may have an effect on the results of this study.

### Conclusion

In conclusion, despite the above imitations, the present meta-analysis indicates that AAC is associated with adverse outcomes, including all-cause mortality and CV events (fatal and non-fatal) in dialysis patients. Our study provides the evidence that detection of AAC is beneficial for risk stratification of dialysis patients with ESRD and has some benefits to clinical diagnosis and treatment processes. In future, more prospective trials with large sample size are needed to further evaluate the relationship between AAC and the prognosis of dialysis patients, especially the impact of the severity of AAC.

## Supporting information

S1 Table PRISMA 2009 checklist. (DOC)

S2 Table Search strategies and detailed records. (PDF)

S3 Table Data for calculation of a simple kappa statistic. (PDF)

S4 Table Raw data of included studies. (PDF)

S1 Fig Funnel plots of CV events (fatal and non-fatal) model. (TIF)

S2 Fig Funnel plots of CV mortality model. (TIF)

S3 Fig Funnel plots of all-cause mortality and CV events model. (TIF)

S4 Fig Sensitivity analysis of all-cause mortality model. (TIF)

S5 Fig Sensitivity analysis of CV events (fatal and non-fatal) model. (TIF)

S6 Fig Sensitivity analysis of CV mortality model. (TIF)

S7 Fig Sensitivity analysis of all-cause mortality and CV events model. (TIF)
