# Supplementary material for: Abdominal aortic calcification can predict all-cause mortality and CV events in dialysis patients: A systematic review and meta-analysis
Source: PLoS One. 2018 Sep 21;13(9):e0204526. doi: 10.1371/journal.pone.0204526 (PMC6150537; doi:10.1371/journal.pone.0204526)
Supplement: S2 Table — (PDF) [file pone.0204526.s002.pdf]

S1 Table. Search strategies and detailed records.

PUBMED:
#1 peritoneal dialysis
#2 Peritoneal Dialyses
#3 Dialysis, Peritoneal
#4 Dialyses, Peritoneal
#5 #1 OR #2 OR #3 OR #4
#6 haemodialysis
#7 hemodialysis
#8 Hemodialyses
#9 #6 OR #7 OR #8
#10 End Stage Renal Disease
#11 Chronic Kidney Failure
#12 End Stage Kidney Disease
#13 ESRD
#14 #10 OR #11 OR #12 OR #13
#15 #5 OR #9 OR #14
#16 Abdominal Aorta
#17 Abdominal Aortic
#18 #16 OR #17
#19 calcification
#20 calcium
#21 #18 AND #20
#22 cohort study
#23 follow up study
#24 follow-up study
#25 followup study
#26 prospective study
#27 retrospective study
#28 outcome
#29 prognosis
#30 mortality
#31 death
#32 predict
#33 #22 OR # 23 OR #24 OR #25 OR #26 OR #27 OR #28 OR #29 OR #30 OR #31 OR #32
#34 #15 AND #21 AND #33

EMBASE:
#1 peritoneal AND (‘dialysis’/exp OR dialysis)
#2 hemodialysis
#3 esrd
#4 end AND stage AND renal AND diaease
#5 abdominal AND aortic
#6 abdominal AND aorta
#7 calcification
#8 calcium
#9 ‘follow up’: ti,ab,kw OR ‘longitudinal’:ti,ab,kw OR ‘outcome’:ti,ab,kw OR ‘prognosis’:ti,ab,kw OR ‘mortality’/exp OR mortality OR ‘death’/exp OR prospective OR retrospective
#10 #5 OR #6
#11 #7 OR #8
#13 #1 OR #2 OR #3 OR #4
#14 #9 AND #12 AND #13

MEDLINE:

S1 peritoneal dialysis OR hemodialysis OR esrd OR end stage renal disease
S2 abdominal aorta OR abdominal aortic
S3 calcification OR calcium
S4 mortality OR death OR follow up OR longitudinal OR outcomes OR prognosis OR prospective OR retrospective OR predict
S5 S2 AND S3
S1 AND S4 AND S5
