# Supplementary material for: Abdominal aortic calcification can predict all-cause mortality and CV events in dialysis patients: A systematic review and meta-analysis
Source: PLoS One. 2018 Sep 21;13(9):e0204526. doi: 10.1371/journal.pone.0204526 (PMC6150537; doi:10.1371/journal.pone.0204526)
Supplement: S3 Table — (PDF) [file pone.0204526.s003.pdf]

S3 Table .data for calculation of a simple kappa statistic

	Reviewer author 2 Yang HONG				
		Include	Exclude	Unsure	Total
Reviewer author 1 Qingyu NIU	Include	9	0	1	10
	Exclude	0	354	4	358
	Unsure	1	1	8	10
	Total	10	355	13	378
