# Supplementary material for: Abdominal aortic calcification can predict all-cause mortality and CV events in dialysis patients: A systematic review and meta-analysis
Source: PLoS One. 2018 Sep 21;13(9):e0204526. doi: 10.1371/journal.pone.0204526 (PMC6150537; doi:10.1371/journal.pone.0204526)
Supplement: S4 Table — (PDF) [file pone.0204526.s004.pdf]

S4 Table. Raw Data of Included Studies

Study/year	Region	Design	Patients	Sample size（% men）	Age **(Mean)**	Detection Methods	Scoring Methods	Comparison of AAC	Events Number HR (95% CI)	Follow-up (months)	Adjustment for Covariates	**Risk of bias according to** ROBINS-I
Makela2018[12]	Sweden, Finland, Denmark, Estonia	Prospective	PD	249 (66.7)	61	Plain lateral lumbar X-ray	AAC-24	Grade3(7-24) VS. Grade2(1-6) VS. Grade1(Absence)	all death (91), 4.85(1.09-21.63), 2.22(0.44-11.18); new CV events (include CV death) 2.59(1.00-6.72); 2.30(0.84-6.34)	46	age, gender, BMI, DM, ABI, ALB	Moderate
Wang2017[23]	China	Prospective	HD	170	NS	lateral abdominal radiograph	AAC-24	High VS. low (AAC≥5 VS. AAC<5)	All death 4.373 (1.562-7.246)			Moderate
Rroji2017[22]	Albania	NS	PD, HD	126	62.6	Lateral lumbar spine radiograph	AAC-24	High VS. low (AAC≥7 VS. AAC<7)	CV death 2.25 (1.77-5.58)			Moderate
NasrAllah2016[9]	Egypt	Prospective	HD	93 (48.3)	42.7	Lateral lumbar spine radiographs	AAC-24	Presence VS. absence	All death (28) 1.2 (0.4–4)	46.8	NS	Moderate
Kwon2014[14]	Korea	Retrospective	HD	112 (43.5)	59	left lumbar spine radiograph	AAC-24	High VS. low (mean AAC 8)	All death (18)4.205 (1.658–10.669); CV events (include CV death) 1.801(1.281–2.531)	32.8	CCI score, ESRD&HD durations, Coronary score, CRP, Ca, LDL, iPTH	Low
Yoon2013[24]	Korea	Retrospective	PD	92 (52.2)	55	abdominal CT scan	ACI	High VS low (mean AAC18.9)	All death (30) and CV events 5.25(1.77 – 15.58)	35.3	age, DM, pre-CVD, HB, ALB, CRP, LAD, ejection fraction	Moderate
Martino2013[20]	Italy	Prospective	PD	72 (60.8)	NS	left lateral plain radiograph	AAC-24	Grade3 (>12) VS Grade2(6-12) VS Grade1(<6)	CV events (include CV death) 0.7(3.562–264.841); 3.918(0.419-36.668)	30.5	age, urine output	Serious
Hong2013[21]	China	Retrospective	HD	217 (49.8)	60	lateral abdominal radiograph	AAC-24	Presence VS. absence	All death (37) 4.47(1.55-12.92) CV death (23) 2.86(0.93-8.81)	26	age, DM, P, ALB, HP, Kt/V, PP	Moderate
Verbeke2011[15]	European	Prospective	PD, HD	1076	61.9	plain lateral lumbar radiograph	AAC-24	Grade3(<5) VS Grade2(5-15) VS Grade1(<5)	All death (234) and CV events 8.640(3.528-21.158); 3.682(1.356-9.997)	24	age, DM, ALB	Moderate
Okuno2007[16]	Japan	cohort study	HD	515 (59.4)	60.1	left lateral abdomen radiograph	AAC-24	Presence VS. absence	All death (103) 2.07(1.21-3.56); CV death (41) 2.39(1.01-5.66)	51	age, HD duration, DM, BMI, ALB, CRE, P, CRP	Moderate
