# Supplementary figures and images for: Abdominal aortic calcification can predict all-cause mortality and CV events in dialysis patients: A systematic review and meta-analysis
Source: PLoS One. 2018 Sep 21;13(9):e0204526. doi: 10.1371/journal.pone.0204526 (PMC6150537; doi:10.1371/journal.pone.0204526)

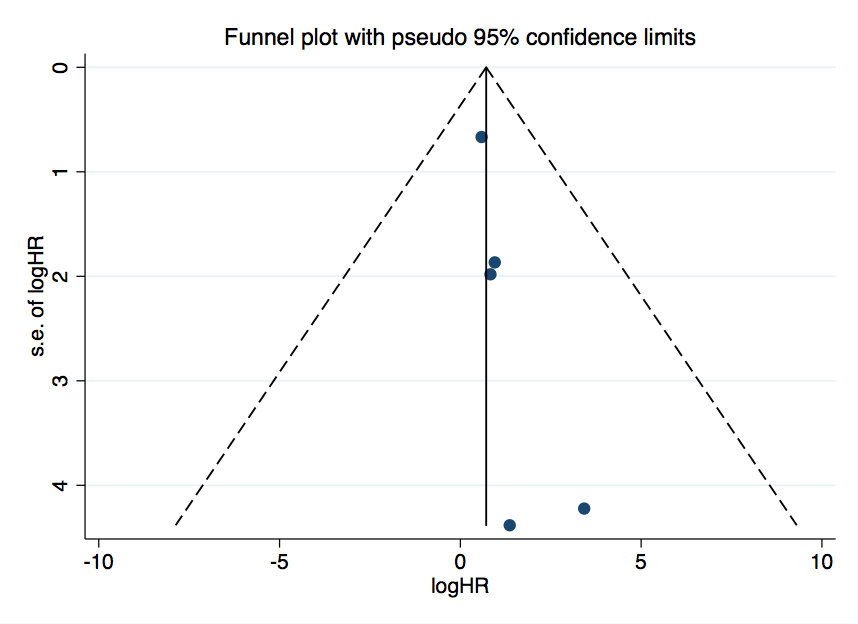

Supplement: S1 Fig — (TIF) [file pone.0204526.s005.tif]

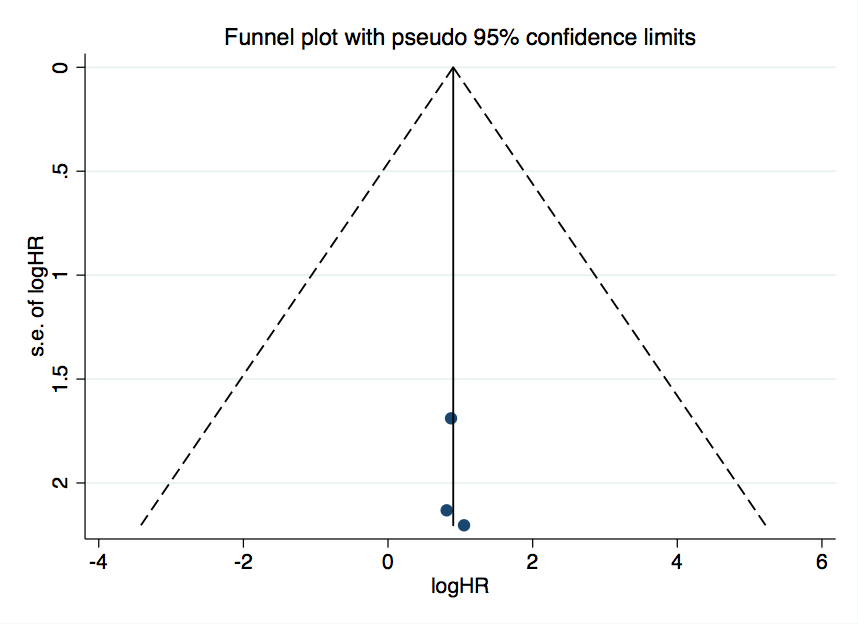

Supplement: S2 Fig — (TIF) [file pone.0204526.s006.tif]

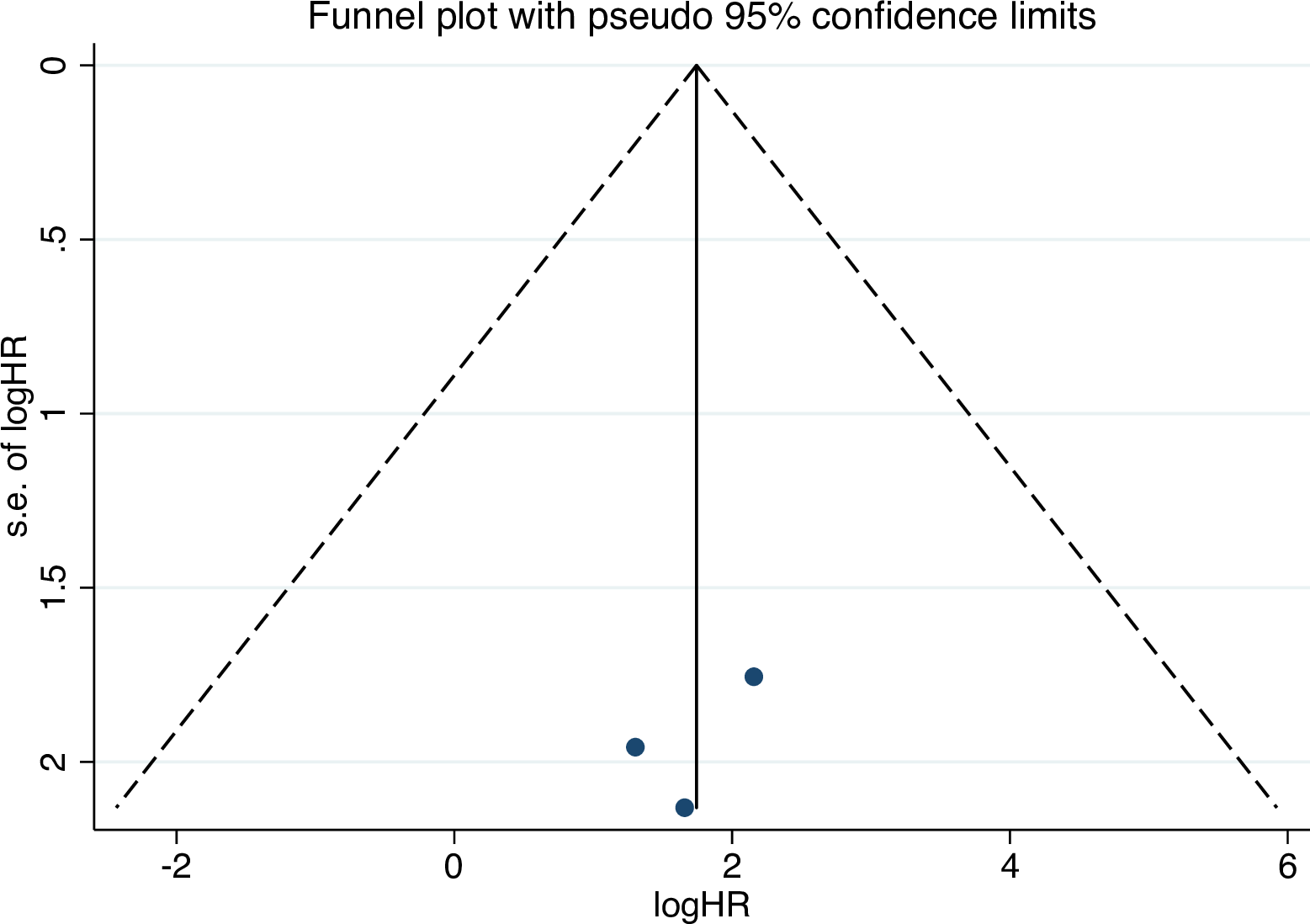

Supplement: S3 Fig — (TIF) [file pone.0204526.s007.tif]

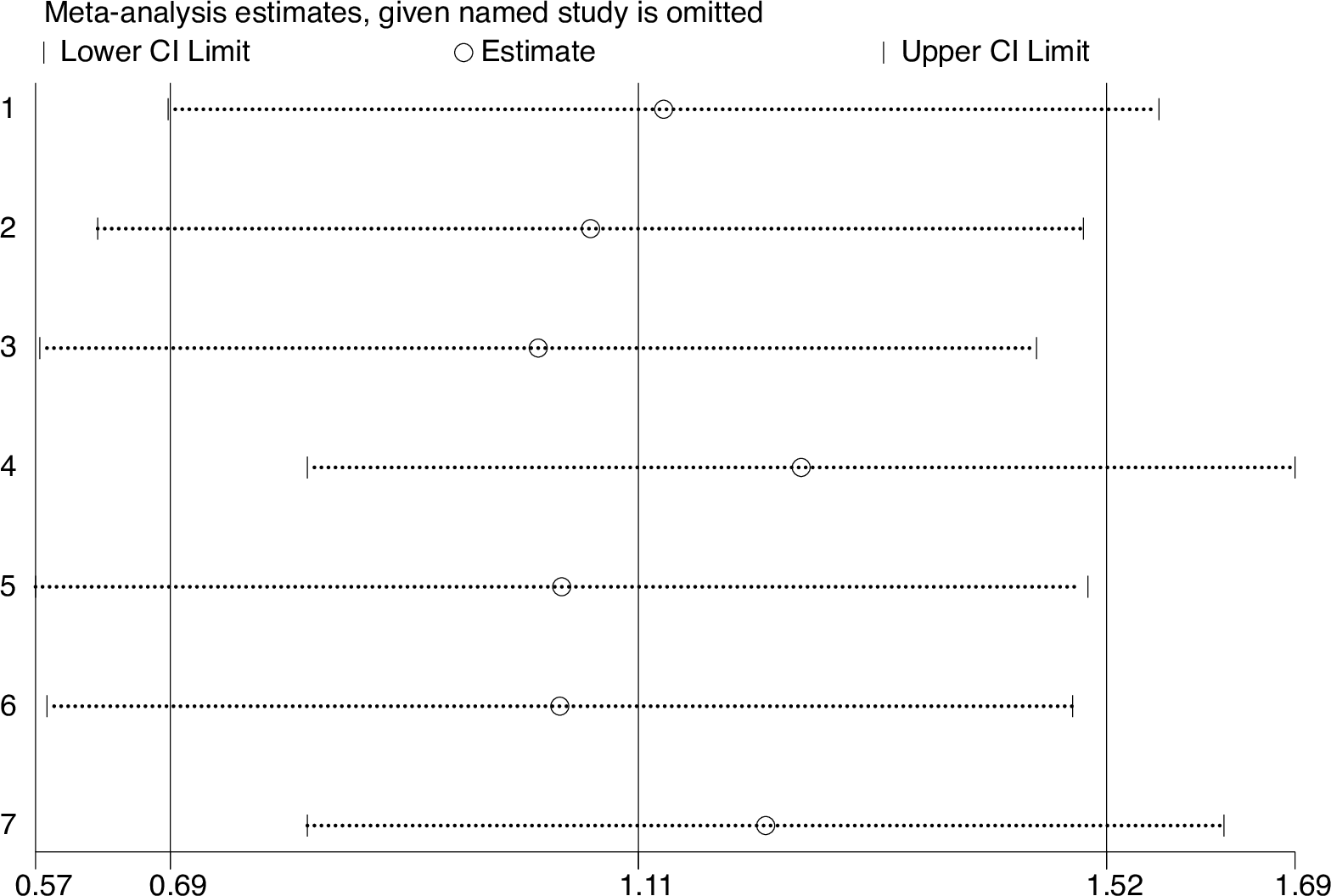

Supplement: S4 Fig — (TIF) [file pone.0204526.s008.tif]

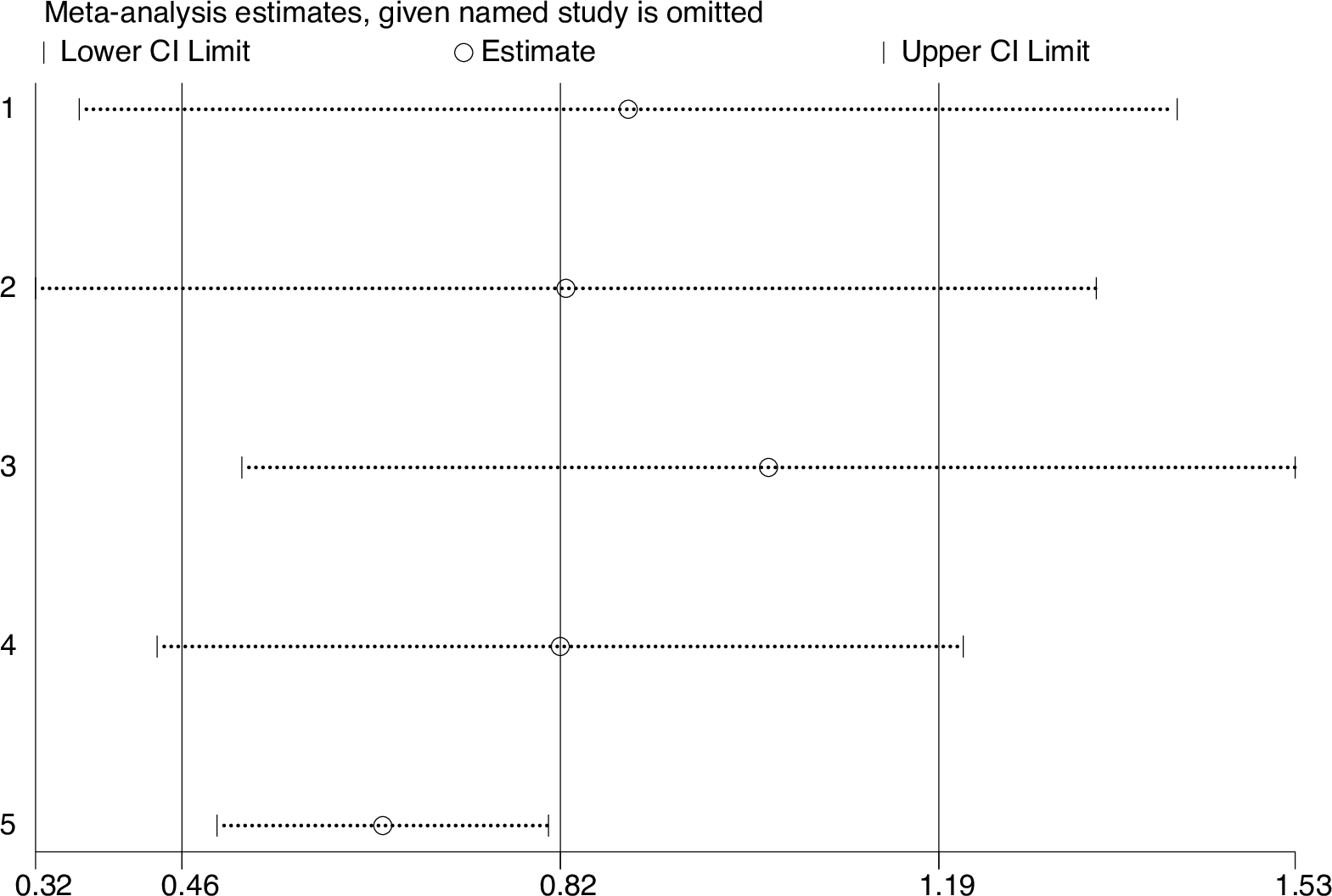

Supplement: S5 Fig — (TIF) [file pone.0204526.s009.tif]

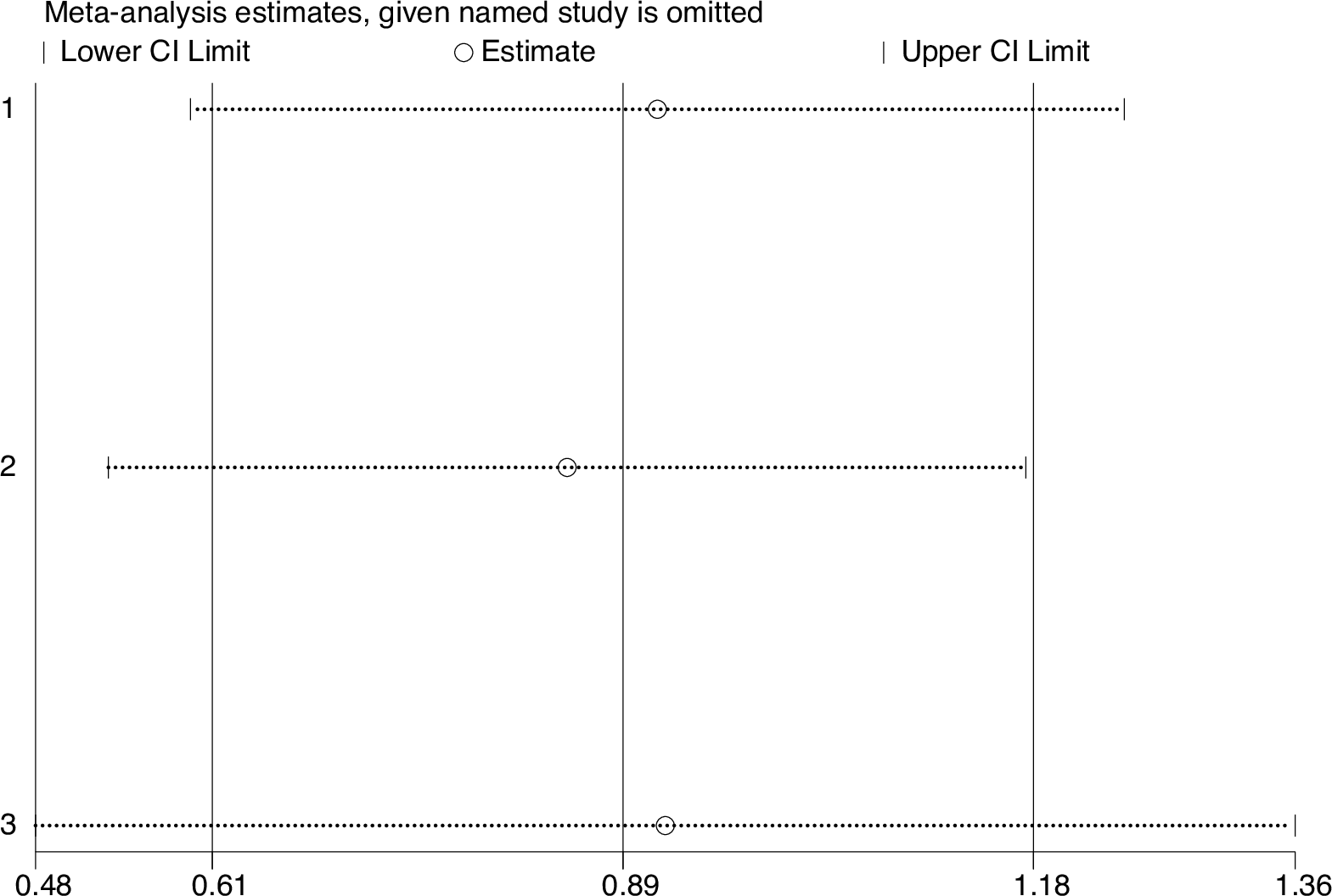

Supplement: S6 Fig — (TIF) [file pone.0204526.s010.tif]

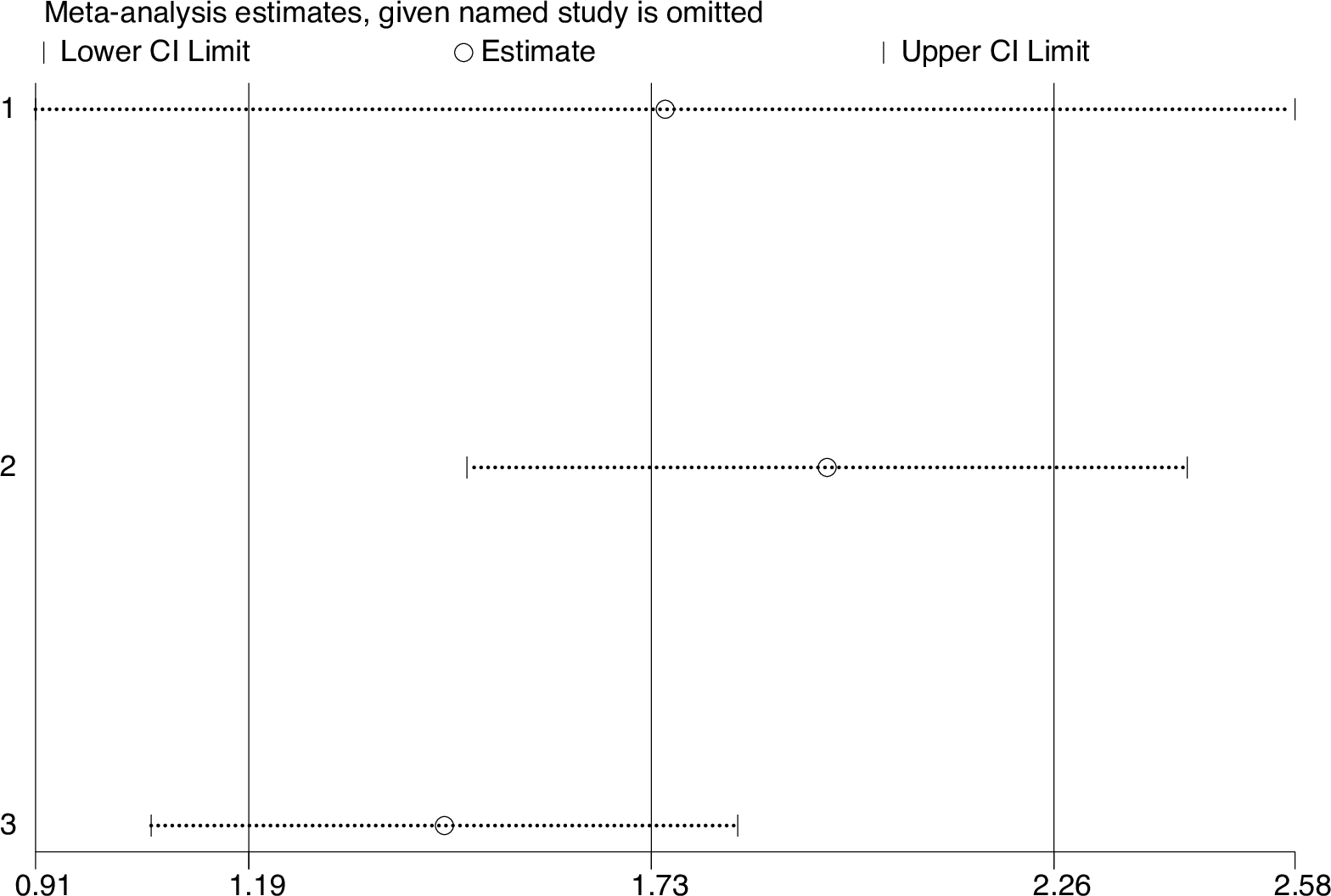

Supplement: S7 Fig — (TIF) [file pone.0204526.s011.tif]
